# Shades of knowledge: awareness among Brazilian dentists on non- and microinvasive approaches for tooth discoloration

**DOI:** 10.1590/1678-7765-2025-0589

**Published:** 2026-02-16

**Authors:** Giovanni Aguirra Liberatti, Karin Landmayer, Bruna de Oliveira Iatarola, Maria Paula Novaes Camargo Manna, Raquel Shimizu Mori, Flávia Pardo Salata Nahsan, Heitor Marques Honório, Luciana Fávaro Francisconi-dos-Rios

**Affiliations:** 1 Universidade de São Paulo Faculdade de Odontologia Departamento de Dentística São Paulo SP Brasil Universidade de São Paulo, Faculdade de Odontologia, Departamento de Dentística, São Paulo (SP), Brasil.; 2 Universidade Federal do Sergipe Faculdade de Odontologia Aracajú SE Brasil Universidade Federal do Sergipe, Faculdade de Odontologia, Aracajú, SE, Brasil.; 3 Universidade de São Paulo Faculdade de Odontologia de Bauru Departamento de Odontopediatria, Ortodontia e Saúde Coletiva Bauru SP Brasil Universidade de São Paulo, Faculdade de Odontologia de Bauru, Departamento de Odontopediatria, Ortodontia e Saúde Coletiva, Bauru, SP, Brasil.

**Keywords:** Tooth discoloration, Tooth bleaching, Resin infiltration, Enamel microabrasion, Dental education

## Abstract

Tooth discoloration is a frequent concern among dental patients. While many professionals and patients choose veneers or other invasive solutions, Minimal Intervention Dentistry (MID) recommends non- and microinvasive approaches to preserve tooth structure.

**Objective:**

To assess Brazilian dentists’ knowledge and practices on tooth discoloration and how education and professional profile may influence them.

**Methodology:**

A cross-sectional observational study was conducted with 384 volunteer dentists via an online series of questions on causes, diagnosis, treatment (per tissue removal), and professional-patient relationship. Answers were scored (1=correct/affirmative, 0=incorrect/negative). Descriptive and multivariate regression analyses were performed (α=0.05).

**Results:**

Most respondents were female, aged 20-49, from the Southeast region. Correct answer rates: 69.9% for causes, 69.4% for diagnosis, 48.8% for treatment, 83.6% for professional-patient relationship, 56.4% overall. Graduation from public universities and holding graduate training in Operative or Pediatric Dentistry or Orofacial Harmonization were associated with better outcomes.

**Conclusion:**

Knowledge of non- and microinvasive approaches to tooth discoloration remains limited. Broader educational efforts are needed, especially among specific groups of professionals who showed lower performance.

## Graphical abstract

**Figure:**
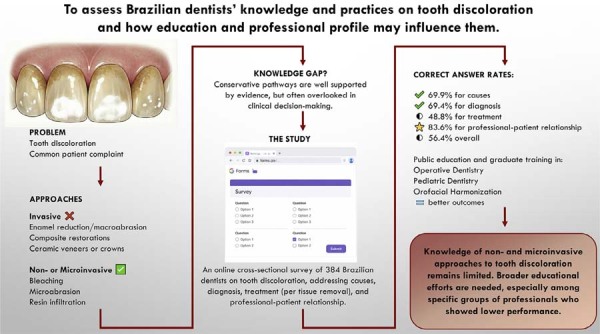

## Introduction

Tooth discoloration is a common complaint in routine dental practice. Social media and mass culture have significantly increased demand for aesthetic procedures to address this condition. Patients dissatisfied with the appearance of discolored or stained teeth often seek dental care to improve the aesthetics of their smile, aiming for greater self-satisfaction and social acceptance.^1,2^ The perception of an individual by others is demonstrably enhanced when the person exhibits “normal” rather than “altered” shades, and aligned rather than misaligned teeth.^2,3^ White spot lesions on anterior teeth are also perceived negatively by the patients themselves, and often lead them to seek treatment for primarily aesthetic rather than therapeutic reasons.^4^

For treatments to be successful, clinicians must be able to accurately identify and assess the specifics of each case of discoloration before recommending the most suitable approach: ideally, one that preserves as much tooth structure as possible,^5^ in accordance with the principles of Minimal Intervention Dentistry (MID).^6^ Proper diagnosis is essential to prevent overtreatment, which also stems from misapplication or misunderstanding of evidence-based criteria, and from knowledge or skill deficits among health professionals.^7^

In treatment planning, aesthetic expectations should be integrated with biological and functional considerations, in a shared decision-making process with the patient.^1^ Patient preferences are not always aligned with the best clinical option; despite this, overtreatment remains a concern in Dentistry, with many clinicians adhering to an outdated and predominantly restorative mindset.^7^ Aesthetic restorations and direct or indirect veneers remain common first-line treatments for discoloration,^5,8^ though they entail enamel and/or dentin removal and are thus considered invasive to varying degrees.^6^ The elective removal of sound tooth structure should be avoided unless a demonstrable long-term benefit outweighs available alternatives.^5,8^

Non-invasive or microinvasive techniques, which involve no or only minimal micrometric enamel removal, may, alone or in combination, provide satisfactory aesthetic outcomes in cases of tooth discoloration.^9^ Depending on the depth and nature of the discoloration, these can include professional cleaning, bleaching, resin infiltration, or microabrasion. Cleaning may be sufficient in cases of extrinsic discoloration due to staining agents or chromogenic interactions on the enamel surface.^10^ Bleaching is the treatment of choice for intrinsic stains when cleaning proves ineffective, particularly in developmental disorders or acquired causes such as aging, pulpal hemorrhage or necrosis, and tetracycline staining.^11^ Whether in-office or at-home, it is a safe and accessible method that acts through the oxidative cleavage of pigment molecules.^10^

Resin infiltration was initially intended to arrest the progression of incipient caries lesions, but has shown favorable aesthetic effects by masking white spot lesions.^12^ Its efficacy is due to the refractive index of the infiltrant approximating that of sound enamel, reducing light scattering and lesion visibility.^13^ The technique has also been proposed for mild-to-moderate fluorosis and hypomineralization, including molar-incisor hypomineralization.^14^ Microabrasion, involving the mechanical application of acidic and abrasive compounds to enamel, is best indicated for superficial stains after bleaching and infiltration have been ruled out. This method removes surface enamel irregularities and alters optical properties, improving aesthetics.^15^

In more complex but enamel-limited stains, non-invasive and microinvasive modalities may be applied sequentially or in a complementary manner. Bleaching reduces chromatic contrast and oxidizes pigments throughout the enamel; microabrasion removes a thin outer layer, facilitating access for subsequent interventions; and resin infiltration masks the remaining whitish areas by modifying light scattering. These choices should always be tailored to the clinical presentation and patient needs.^9^ Despite strong evidence supporting this conservative pathway, many clinicians still bypass it and proceed directly to invasive (even minimally invasive) procedures, such as enamel reduction/macroabrasion, composite restorations, or ceramic veneers or crowns, resulting in avoidable removal of sound tooth structure. This tendency likely reflects the limited translation of MID principles to the field of tooth discoloration, where structured decision-making guidance remains scarce. The pursuit of the ideal smile must not override the commitment to evidence-based, conservative care that prioritizes oral health and structural preservation.^7^

In light of the gap between current clinical practices and scientific evidence, this study aimed to evaluate Brazilian dentists’ knowledge and clinical approaches regarding tooth discoloration, with a focus on non- and microinvasive strategies. Additionally, the study sought to investigate how factors related to professional profile and education influence their understanding and decision-making on the topic. It was hypothesized that overall knowledge would be below good, and that specific professional profiles would demonstrate better performance than others.

## Methodology

### Study design and ethical aspects

This cross-sectional, observational, and analytical study was conducted via an online series of questions distributed to Brazilian dentists. The objective was to assess their knowledge of tooth discoloration, their clinical management strategies, and how their educational and professional backgrounds may influence their decision-making processes.

The series of questions was designed explicitly for this study, which was approved by the local Human Research Ethics Committee (CAAE: 69734823.0.0000.0075; approval numbers: 6.104.048, 6.555.094, and 8.023.962). Dentists were invited to participate by a formal invitation containing a link to the online platform (Google Forms). Upon accessing the link, they were first presented with an Informed Consent Form (ICF) for thorough review, which provided all necessary information regarding the study’s objectives, procedures, investigators, and ethical considerations. Only after affirmatively accepting the consent, considering their electronic signature, were participants directed to the instructions for completing the series of questions and subsequently to the questions themselves.

Participation was entirely voluntary and unpaid. All responses were collected anonymously and handled confidentially. Email collection and the option to send response copies were disabled in the platform settings to ensure anonymity. No cookies or IP tracking were used. Participants could exit the questionnaire at any time before submission simply by closing the browser; as a result, no data on refusals were collected, and specific reasons for non-participation could not be determined. Due to the handling of anonymous data, once submitted, responses could not be withdrawn. Nevertheless, for each question, participants could select “Prefer not to answer”. Respondents were encouraged to save a PDF copy of the ICF for their own records. There were no direct benefits to participation, and associated risks were minimal, limited to the time commitment, the use of a device with internet access, and potential inconveniences inherent to the online format. The study did not receive external funding and was conducted using only personal or institutional resources.

### Development and characteristics of the series of questions

Because, to the best of current knowledge, no professional society guidelines address how the concepts of non- and microinvasive care should be applied to tooth discoloration, and the topic has only been preliminarily explored in a single published review,^9^ the series of questions relied on that literature to provide the most coherent framework currently available. That review is grounded in the established caries consensus,^6^ which defines levels of invasiveness within MID. A preliminary version of the instrument was then developed in Google Forms and underwent content validity assessment by 10 dentists (2 general practitioners, 5 graduate students, and 3 faculty members, all affiliated with the authors’ professional network and varying in familiarity with the subject). These professionals evaluated the clarity and objectivity of the questions and response options, the relevance of the content covered, the need for additional items, and the estimated time required for completion.

Following their feedback, an improved version of the series of questions (unvalidated English version available as Appendix 1) was finalized, consisting of two sections. Section 1 (questions 1.1 to 1.8) collected data on respondents’ demographic, professional, and educational backgrounds. Section 2 (questions 2.1 to 2.25) assessed their understanding of tooth discoloration and related non-invasive and microinvasive treatment options. Specifically, questions 2.1 to 2.5 addressed the causes of tooth discoloration; 2.6 to 2.8 focused on diagnostic approaches; 2.9 to 2.22 explored treatment strategies and the amount of tissue removed; and 2.23 to 2.25 examined the professional-patient relationship in context and decision-making priorities. Each question was designed with reference to the current scientific literature, including predetermined response options and a single correct answer. For questions 2.23 and 2.24, responses were classified as affirmative or negative, without a predefined correct option. For question 2.25, only the distribution of responses was analyzed.

### Sample size calculation

The sample size was estimated considering a population of approximately 400,000 dentists (394,655 registered dentists with active licenses; latest update from the Federal Council of Dentistry [CFO], May 8, 2023; https://website.cfo.org.br/estatisticas/ quantidade-geral-de-entidades-e-profissionais-ativos/; accessed May 8, 2023), an expected prevalence of 50%, a 4% margin of error, and a 95% confidence interval. This yielded a minimum of 384 participants, ensuring 95.5% power to detect a prevalence ratio of 1.3 at a 5% significance level.

### Sample acquisition

Eligible participants were dentists aged ≥18 years, residing in Brazil, who voluntarily agreed to participate after providing informed consent. Recruitment was conducted by professional associations and societies (Federal Council of Dentistry - CFO, São Paulo Regional Council of Dentistry - CROSP, and the Brazilian Group of Operative Dentistry Professors - GBPD), universities, and social media platforms (Facebook and Instagram, with sponsored posts). Additional invitations were sent individually via email or WhatsApp, without disclosure of personal contact information to third parties.

All invitations included study details and a link to the online series of questions (Google Forms^®^). Upon accessing the link, participants were first directed to the informed consent form, which they were required to read in full. Only after explicitly selecting an affirmative response to the consent statement (considered equivalent to signing the form) were participants granted access to the instructions and survey questions. Data collection continued until 384 completed responses were obtained, extending from June 2023 to March 2024.

### Eligibility criteria

Inclusion criteria comprised licensed dentists with clinical experience as general practitioners and/or specialists, as well as dentists engaged in teaching at the undergraduate or graduate level in Dentistry. Exclusion criteria were: professionals in the dental field who were not licensed dentists; dentists who had graduated but never practiced; dentists teaching in disciplines unrelated to undergraduate or graduate Dentistry programs; individuals under 18 years of age; individuals considered vulnerable; and those who did not provide informed consent.

### Data interpretation

After data collection, responses were exported to Excel spreadsheets and stored locally on a secure electronic device.

Responses were coded as 1 for correct or affirmative answers and 0 for incorrect or negative answers. Outcomes were calculated as the percentage of correct responses for each group of questions and for the overall set of the first three groups: (i) perspective on the causes of tooth discoloration (Q2.1-2.5); (ii) methods used to assess it (Q2.6-2.8); (iii) treatment options and amount of tissue removed (Q2.9-2.22); (iv) professional-patient relationship in context (Q2.23-2.24); and (v) overall expertise on the topic (Q2.1-2.22). For Q2.25, only the distribution of responses was analyzed.

Potential predictors included the following respondent characteristics: gender (male, female, not reported); region of residence (Southeast, South, North, Midwest, Northeast, not reported); age group (20-29, 30-39, 40-49, 50-59, 60-69, ≥70 years, not reported); years since graduation (<1, 1-3, 4-10, 11-20, >20 years, not reported); type of undergraduate institution (public, private, not reported); holding a lato sensu graduate degree (no, yes, in progress, not reported); holding a lato sensu graduate degree in Operative Dentistry, Pediatric Dentistry, or Orofacial Harmonization (no, yes, not reported); holding a stricto sensu graduate degree (no, yes, in progress, not reported) and highest level attained (postdoctoral, doctoral, master’s, none, not reported); holding a stricto sensu graduate degree in Operative Dentistry, Pediatric Dentistry, or Orofacial Harmonization (no, yes, not reported); and current field of practice (teaching, clinical practice, both, not reported).

### Statistical analysis

Descriptive analyses were performed for all predictors and outcomes. Outcome performance was categorized as minimal (0-20%), poor (20.1-40%), moderate (40.1-60%), good (60.1-80%), or excellent (80.1-100%).

Bivariate regressions were conducted for all predictors, and those with p < 0.20 were included in multiple linear regression models, as this more flexible threshold is standard practice to avoid excluding potentially relevant variables before multivariable adjustment. A stepwise backward procedure was then used to retain only significant predictors in the final models. The impact of each predictor on the outcomes was ultimately assessed by first examining the model’s F, p, and adjusted R^2^ values, followed by the p-values and β coefficients for each predictor level compared to the reference level (the condition most likely to produce response errors).

Descriptive analyses were performed with Statistica 10.0 (TIBCO Software Inc., Palo Alto/CA, USA), and regression analyses with Jamovi 2.3 (https://www.jamovi.org). Significance was set at 5%, except for bivariate analyses.

## Results

A total of 384 Brazilian dentists who provided informed consent completed the online series of questions. Most respondents were female (63.5%), from the Southeast region (91.1%), and aged 20-29 (25.3%), 30-39 (26.6%), or 40-49 (20.6%) years. Over one-third had been practicing dentistry for more than 20 years (36.2%), held a degree from a public institution (59.6%), and held a lato sensu graduate degree (64.8%), primarily outside Operative Dentistry, Pediatric Dentistry, or Orofacial Harmonization (78.4%). The majority did not hold a stricto sensu degree (57.3%) nor in the above specialties (80.7%). Most worked in private clinics (70.6%). Absolute and relative frequencies for each sociodemographic, educational, and practice characteristic are detailed in Tables 1 [Table t2]-3.

**Table 1:** Absolute and relative frequencies for each category of the respondents’ sociodemographic characteristics.

		n	%
Gender	Male	137	35.7
	Female	244	63.5
	Prefer not to answer	3	0.8
Region	Southeast	350	91.1
	South	8	2.1
	Northeast	13	3.4
	Midwest	5	1.3
	North	5	1.3
	Prefer not to answer	3	0.8
Age group	20-29	97	25.3
	30-39	102	26.6
	40-49	79	20.6
	50-59	58	15.1
	60-69	43	11.2
	70 or older	5	1.3
	Prefer not to answer	0	0

**Table 2 t2:** Absolute and relative frequencies for each category of the respondents’ educational characteristics.

		n	%
Professional Experience	Less than 1 year	10	2.6
	1-3 years	54	14.1
	4–10 years	114	29.7
	11–20 years	67	17.4
	More than 20 years	139	36.2
	Prefer not to answer	0	0.0
Higher Education Institution	Public	229	59.6
	Private	153	39.8
	Prefer not to answer	2	0.5
Lato Sensu Graduate Education	Yes	249	64.8
	No	91	23.7
	In progress	42	10.9
	Prefer not to answer	2	0.5
Lato Sensu Graduate Education in Operative Dentistry, Pediatric Dentistry, or Orofacial Harmonization	Yes	83	21.6
	No	301	78.4
Stricto Sensu Graduate Education	Yes	140	36.5
	No	220	57.3
	In progress	22	5.7
	Prefer not to answer	2	0.5
Highest Level of Graduate Education	Master’s degree	52	13.5
	Doctorate (PhD)	91	23.7
	Postdoctoral training	17	4.4
	None / Did not answer	224	58.3
Stricto Sensu Graduate Education in Operative Dentistry, Pediatric Dentistry, or Orofacial Harmonizati	Yes	74	19.3
	No	310	80.7

**Table 3:** Absolute and relative frequencies for each category of the respondents’ primary workplace location.

		%	n
Primary Workplace	Academic/Teaching	41	10.7
	Clinical Practice	271	70.6
	Both	50	13.0
	Other	14	3.6
	Prefer not to answer	8	2.1

Respondents correctly identified the causes of dental discoloration as follows: external discoloration from smoking and exposure to colorants, 66.7% (good); internal developmental defects (imperfect dentinogenesis/amelogenesis, fluorosis, enamel hypoplasia/hypomineralization), 80.5% (excellent); pulpal hemorrhage/necrosis causing black ferrous sulfate formation, 73.7% (good); tetracycline-induced discoloration via calcium chelation during tooth development, 70.1% (good); and incipient smooth-surface caries affecting enamel translucency, 58.9% (moderate). These findings suggest lower familiarity with the aesthetic impact of caries lesions compared to other causes of dental discoloration.

Regarding the best method for estimating stain depth, 75.8% of respondents correctly identified transillumination (good). For transilluminated stains, 59.1% recognized that darker, well-defined shadows with diffuse margins indicate greater depth (moderate). For discolorations associated with enamel porosity, 73.2% correctly indicated air-drying, which displaces saliva or water from the pores and enhances stain visibility (good). These results highlight that estimating stain depth via transillumination remains the most challenging aspect of stain assessment.

Concerning tissue removal, 58.6% of respondents classified bleaching as non-invasive (moderate), 33.9% classified resin infiltration as microinvasive (poor), 51.3% classified microabrasion as microinvasive (moderate), 46.6% classified ultra-thin ceramic veneers as minimally invasive (moderate), and 80.5% classified traditional laminates as invasive (excellent). When asked to rank treatments from most to least conservative and from least to most predictable, only 34.6% responded correctly (poor). Correct identification rates for treatment indications were as follows: professional cleaning for discolorations from smoking or colorants, 39.6% (poor); bleaching for all listed indications, 23.4% (poor); resin infiltration for enamel porosities due to developmental defects of enamel, 53.1% (moderate); and microabrasion for discolorations of confined enamel or caries lesions only if arrested, 56.5% (moderate). Bleaching was correctly identified as the first-line option for discolored teeth by 60.2% of respondents (good). For resin infiltration, 37.0% correctly recognized that it is not true that the technique provides less satisfactory aesthetic outcomes than fluoride-enhanced remineralization (poor). For enamel microabrasion, 58.6% correctly indicated that it is not true that it has no effect on enamel optical properties (moderate). For complex but enamel-limited discolorations, 49.2% acknowledged the possibility of combining treatments, with bleaching reducing contrast, microabrasion removing the outer surface, and resin infiltration filling residual porosity (moderate). Overall, microinvasive and minimally invasive concepts were less familiar than non-invasive and invasive, and resin infiltration was less well understood than bleaching and microabrasion.

Nevertheless, 85.2% of respondents stated that patients understand when informed that more conservative options exist for managing dental discoloration, even if they initially seek restorations or veneers (excellent). Similarly, 82.0% reported indicating non- or microinvasive treatments (excellent). These findings contrast with the low proportion of correct responses to questions 2.9-2.22, particularly in some items.

Table 4 shows the frequencies of correct and incorrect responses for questions 2.1-2.22, and of affirmative and negative responses for questions 2.23-2.24, with corresponding performance levels.

**Table 4:** Absolute and relative frequencies of correct and incorrect responses for questions 2.1-2.22, affirmative and negative responses for questions 2.23-2.24, and the corresponding performance levels.

Question	Correct/Affirmative		Incorrect/Negative		Performance
	n	%	n	%	
2.1	256	66.7	128	33.3	good
2.2	309	80.5	75	19.5	excellent
2.3	283	73.7	101	26.3	good
2.4	269	70.1	115	29.9	good
2.5	226	58.9	158	41.1	moderate
2.6	291	75.8	93	24.2	good
2.7	227	59.1	157	40.9	moderate
2.8	281	73.2	103	26.8	good
2.9	225	58.6	159	41.4	moderate
2.10	130	33.9	254	66.1	poor
2.11	197	51.3	187	48.7	moderate
2.12	179	46.6	205	53.4	moderate
2.13	309	80.5	75	19.5	excellent
2.14	133	34.6	251	65.4	poor
2.15	152	39.6	232	60.4	poor
2.16	90	23.4	294	76.6	poor
2.17	204	53.1	180	46.9	moderate
2.18	217	56.5	167	43.5	moderate
2.19	231	60.2	153	39.8	good
2.20	142	37.0	242	63.0	poor
2.21	225	58.6	159	41.4	moderate
2.22	189	49.2	195	50.8	moderate
2.23	327	85.2	57	14.8	excellent
2.24	315	82.0	69	18.0	excellent

Preservation of tooth structure was reported as the primary consideration by 57.3% of respondents when selecting a treatment for dental discoloration (Table 5), consistent with the principles of Minimal Intervention Dentistry.

**Table 5:** Absolute and relative frequencies of responses to question 2.25: “What do you prioritize when recommending a specific treatment for dental discoloration?”

	n	%
Preservation of tooth structure	220	57.3
Restoration of tooth form and function	23	6.0
Restoration of smile aesthetics	39	10.2
Longevity of the proposed treatment	9	2.3
Personal satisfaction	2	0.5
Patient satisfaction	38	9.9
Patient health	36	9.4
Fees to be received	1	0.3
Other(s)	2	0.5
Prefer not to answer	0	0

Respondents’ performance varied across outcomes: 69.9% correct responses for perspective on the causes of tooth discoloration and 69.4% for methods used to assess it (both good), 48.8% for treatment options and amount of tissue removed (moderate), 83.6% for acceptance and recommendation of microinvasive treatments within the professional-patient context (excellent), and 56.4% for overall expertise on the topic (moderate) (Table 6).

**Table 6:** Mean, median, first and third quartiles (25 and 75%), standard deviation (SD), and performance scores for correct or affirmative responses for each outcome, along with the corresponding performance level.

Outcomes	Mean	SD	Median	25%	75%	Performance
Perspective on the causes of tooth discoloration	0.699	0.247	0.800	0.600	0.800	good
Methods used to assess it	0.694	0.297	0.667	0.333	1.000	good
Treatment options and amount of tissue removed	0.488	0.160	0.500	0.429	0.571	moderate
Professional-patient relationship in context	0.836	0.314	1.000	1.000	1.000	excellent
Overall expertise on the topic	0.564	0.152	0.591	0.455	0.682	moderate

Regarding the multiple linear regression for the outcome “perspective on the causes of tooth discoloration”, the analysis yielded a statistically significant model (F=7.32; p<0.001; Adjusted R^2^=0.117), with age group, type of undergraduate institution, and holding a stricto sensu graduate degree in Operative Dentistry, Pediatric Dentistry, or Orofacial Harmonization as potential predictors (Table 7). Assumption testing indicated p<0.05 only for normality, but not for heteroscedasticity, autocorrelation, or collinearity. Correct response rates were higher among respondents aged 20-29 (+26.3%), 30-39 (+29.6%), 40-49 (+27.5%), 50-59 (+29.5%), and 60-69 years (+27.8%) compared to those ≥70 years, graduates from public institutions (+10.8%) compared to private institution graduates, and holders of stricto sensu graduate degrees in Operative Dentistry, Pediatric Dentistry, or Orofacial Harmonization (+14.0%) compared to those without.

**Table 7:** Final multiple linear regression model for the outcome “perspective on the causes of tooth discoloration”.

	β coefficient	t	p
Intercept ^a^	0.332	3.11	0.002
Age group:			
20-29 vs. 70+	0.263	2.46	0.014
30-39 vs. 70+	0.296	2.77	0.006
40-49 vs. 70+	0.275	2.55	0.011
50-59 vs. 70+	0.295	2.71	0.007
60-69 vs. 70+	0.278	2.50	0.013
Undergraduate Institution:			
Public vs. Private	0.108	4.17	< .001
X vs. Private	-0.219	-1.32	0.188
Stricto Sensu - Operative Dentistry, Pediatric Dentistry, or Orofacial Harmonization? Yes vs. No	0.140	4.58	< .001

^a^ Represents the reference level

For the outcome “methods used to assess dental discoloration”, multiple linear regression also produced a statistically significant model (F=7.19; p<0.001; Adjusted R^2^=0.0885), with type of undergraduate institution, holding a lato sensu graduate degree in Operative Dentistry, Pediatric Dentistry, or Orofacial Harmonization, and the highest level of stricto sensu graduate education as potential predictors (Table 8). Assumption testing indicated p<0.05 for normality and heteroscedasticity, albeit not for autocorrelation or collinearity. Correct response rates were higher among respondents who graduated from public institutions (+11.22%) compared to those from private institutions, among respondents holding a lato sensu graduate degree (+7.78%) compared to those without, and among respondents with postdoctoral (+17.22%) or doctoral degrees (+11.20%) compared to those holding a master’s degree.

**Table 8:** Final multiple linear regression model for the outcome “methods used to assess tooth discoloration”.

	β coefficient	t	p
Intercept ^a^	0.5707	12.702	< .001
Undergraduate Institution:			
Public vs. Private	0.1122	3.674	< .001
X vs. Private	-0.2427	-1.197	0.232
Lato Sensu - Operative Dentistry, Pediatric Dentistry, or Orofacial Harmonization?			
Yes vs. No	0.0778	2.183	0.030
Highest degree:			
Doctorate vs. Master’s	0.1120	2.252	0.025
Postdoctoral vs. Master’s	0.1722	2.160	0.031
X vs. Master’s	0.0108	0.244	0.807

^a^ Represents the reference level

For the outcome “treatment options and amount of tissue removed”, multiple linear regression likewise produced a statistically significant model (F=13.70; p<0.001; Adjusted R^2^=0.0906), with type of undergraduate institution and holding a stricto sensu graduate degree in Operative Dentistry, Pediatric Dentistry, or Orofacial Harmonization as potential predictors (Table 9). Assumption testing indicated p<0.05 for normality, heteroscedasticity, and autocorrelation, but not for collinearity. Correct response rates were higher among respondents who graduated from public institutions (+5.96%) than among those who graduated from private institutions, and among those holding relevant stricto sensu graduate degrees in Operative Dentistry, Pediatric Dentistry, or Orofacial Harmonization (+9.11%) than among those without.

**Table 9:** Final multiple linear regression model for the outcome “treatment options and and amount of tissue removed”.

	β coefficient	t	p
Intercept ^a^	0.4350	34.626	< .001
Undergraduate Institution:			
Public vs. Private	0.0596	3.711	< .001
X vs. Private	-0.0421	-0.389	0.698
Stricto Sensu - Operative Dentistry, Pediatric Dentistry, or Orofacial Harmonization?			
Yes vs. No	0.0911	4.573	< .001

^a^ Represents the reference level

For the outcome “professional-patient relationship in context (acceptance and recommendation of microinvasive treatments for dental discoloration)”, the regression model was again statistically significant (F=2.69; p=0.003; Adjusted R^2^=0.0423), with age group, years since graduation, and holding a lato sensu graduate degree in Operative Dentistry, Pediatric Dentistry, or Orofacial Harmonization as potential predictors (Table 10). Assumption testing indicated p<0.05 for normality and heteroscedasticity, but not for autocorrelation or collinearity. Acceptance/recommendation rates were higher among respondents aged 20-29 (+32.1%), 30-39 (+37.6%), 40-49 (+22.7%), 50-59 (+26.5%), and 60-69 years (+25.4%) compared to those ≥70 years. Rates decreased with shorter professional experience, specifically -13.7% (0-1 year), -7.7% (1-3 years), -17.1% (4-10 years), and -12.6% (11-20 years) compared to >20 years. Respondents holding a lato sensu graduate degree in Operative Dentistry, Pediatric Dentistry, or Orofacial Harmonization had a 13.1% higher rate than those without.

**Table 10:** Final multiple linear regression model for the outcome “professional-patient relationship in the context of acceptance and recommendation of microinvasive treatments for dental discoloration”.

	β coefficient	t	p
Intercept ^a^	0.5990	4.339	< .001
Age group:			
20-29 vs. 70+	0.3206	2.049	0.041
30-39 vs. 70+	0.3756	2.472	0.014
40-49 vs. 70+	0.2269	1.586	0.114
50-59 vs. 70+	0.2649	1.845	0.066
60-69 vs. 70+	0.2538	1.745	0.082
Professional experience:			
01–03 years vs. More than 20 years	-0.0770	-0.900	0.369
04–10 years vs. More than 20 years	-0.1714	-2.232	0.026
11–20 years vs. More than 20 years	-0.1264	-1.922	0.055
Less than 1 year vs. More than 20 years	-0.1365	-1.103	0.271
Lato Sensu - Operative Dentistry, Pediatric Dentistry, or Orofacial Harmonization?			
Yes vs. No	0.1314	3.438	< .001

^a^ Represents the reference level

Finally, for the outcome “overall expertise on the topic”, the analysis further produced a statistically significant model (F=25.00; p<0.001; Adjusted R^2^=0.158), with type of undergraduate institution and holding a stricto sensu graduate degree in Operative Dentistry, Pediatric Dentistry, or Orofacial Harmonization as potential predictors (Table 11). Assumption testing indicated p<0.05 for normality, heteroscedasticity, and autocorrelation, but not for collinearity. Correct response rates were higher among respondents who graduated from public institutions (+7.78%) compared to private institutios, and among those holding a lato sensu graduate degree in Operative Dentistry, Pediatric Dentistry, or Orofacial Harmonization (+10.51%) compared to those without.

**Table 11:** Final multiple linear regression model for the outcome “overall expertise on the topic”.

	β coefficient	t	p
Intercept ^a^	0.4979	43.38	< .001
Undergraduate Institution:			
Public vs. Private	0.0778	5.30	< .001
X vs. Private	-0.1116	-1.13	0.261
Stricto Sensu - Operative Dentistry, Pediatric Dentistry, or Orofacial Harmonization?	0.1051	5.77	< .001
Yes vs. No	0.4979	43.38	< .001

^a^ Represents the reference level

## Discussion

As hypothesized, overall knowledge of dental discoloration and its management was moderate, generally with higher performance observed among graduates of public universities and individuals with graduate training in Operative Dentistry, Pediatric Dentistry, or Orofacial Harmonization.

These findings are particularly relevant given the rising demand for aesthetic treatments involving discolored or stained teeth.^1,2^ Despite heightened aesthetic expectations, many practitioners, consistent with patterns observed among pediatric dentists in other settings such as Turkey,^16^ continue to struggle with selecting the most effective and conservative modality for each case.

Diagnostic challenges were evident: at least 30.1% of respondents showed difficulty identifying etiologies of discoloration, and 41.1% struggled specifically with caries white spot lesions. Tooth color varies physiologically from white to cream-yellow and darkens with age and/or due to intrinsic and extrinsic pigment modifications.^17^ Enamel modulates dentin-derived color; reduced thickness increases dentin show-through, while reduced mineral content increases light scattering and opacity, both altering shade perception.^18^ Respondents strongly associated enamel developmental defects with aesthetic impairment but were less sensitive to the visual significance of early caries lesions, reflecting a clinical mindset predominantly centered on preventing cavitation.^19^ Importantly, fluoride-mediated lesion arrest does not inherently restore translucency^20^; thus, combining lesion arrest with aesthetic masking may be desirable for acceptable outcomes.^12,13,20,21^ Enamel porosity, whether from acid-induced demineralization or developmental defects, disrupts light transmission, producing white opacity that limits dentin visibility and affects satisfaction.^22^

Assessing depth and porosity characteristics is essential for treatment selection,^9^ yet 30.6% of respondents had difficulty with this assessment, increasing to 40.9% when using transillumination. Technical complexities of this method are well-established.^23,24^ Transillumination directs light through the lingual or palatal surface, with deeper hypomineralized areas appearing darker and superficial regions transmitting light more readily.^22,25,26^ Heterogeneity in mineral content and lesion depth further complicates assessment, as mechanical properties, mineral density, chemical composition, and optical behavior vary within the lesion and at its interface with sound enamel.^26,27^ Accuracy also depends on light intensity and operator technique. Effectively applying the method requires familiarity and careful handling.^23^ These factors suggest that the observed difficulties likely reflect the practical challenges of mastering the technique.

Treatment planning should therefore follow MID principles,^6^ emphasizing preservation of tooth structure and limiting unnecessary removal. While restorations, veneers, or crowns may sometimes be required, conservative approaches frequently achieve aesthetic restoration while preserving tissue.^9^ Strikingly, over half of respondents (51.2%) reported uncertainty regarding the spectrum of treatment options and their relative tissue impact, demonstrating a critical educational deficit.

In caries management, non-invasive approaches preserve dental tissue entirely, microinvasive techniques remove only micrometric amounts (typically via acid), and mechanically driven interventions are considered invasive, even if minimally so.^6^ Applied to dental discoloration or staining, bleaching is non-invasive, whereas resin infiltration and enamel microabrasion are microinvasive.^9^ Bleaching is the least invasive but least predictable, microabrasion is the most invasive yet most predictable, and resin infiltration falls in between. Ultra-thin indirect veneers (“contact lenses”) are minimally invasive, whereas conventional veneers are clearly invasive.

Notably, to current knowledge, only a single review,^9^ published in Brazil, explicitly frames discoloration treatment according to tissue removal, paralleling established criteria for caries management.^6^ The São Paulo Association of Dentists (APCD) Journal, which published this review, is a long-standing and respected quarterly periodical (77+ years), indexed in BBO, LILACS, and Latindex. Its editorial content integrates clinical perspectives with scientific articles for both general practitioners and specialists, alongside sections on professional development, information dissemination, and the promotion of clinical excellence. Some respondents may have encountered this specific review.^9^ Still, most appeared unfamiliar with it despite residing predominantly in São Paulo and the Southeast region, where access to such material would be expected.

When considering treatment options for discolored or stained teeth, the historical evolution of techniques provides important context. Bleaching with hydrogen peroxide was first documented in the 1920s, with broader adoption following the introduction of carbamide peroxide in the 1960s,^28^ and enamel microabrasion emerged in the late 1980s.^29^ These approaches are well-established and widely recognized by practitioners. In contrast, newer methods such as resin infiltration, which emerged in the late 2000s with Icon^®^ (DMG Chemisch-Pharmazeutische Fabrik GmbH, Hamburg, Germany),^30^ are less familiar, reflecting the gradual incorporation of innovations into everyday clinical practice. Adoption of newer, evidence-based modalities occurs over time, guided by the dissemination of scientific knowledge, continuing professional education, and integration into routine practice.^31^

Survey responses indicate that only about 20% of dentists did not report that their patients understand, when properly explained, that conservative options such as non- or microinvasive treatments exist, even if they initially request a restoration or veneer. Respondents may have followed the expected response or genuinely practiced conservatively, having internalized the prevention and preservation of tooth structure. For many, this mindset guides clinical decisions almost automatically.^32^ Nearly 60% ranked tooth preservation as their primary criterion when selecting treatment, despite multiple alternatives. Applying preventive care, or MID, almost automatically, without deliberate consideration, likely reflects the pressures of a demanding clinical workload and competing priorities, rather than gaps in knowledge, and also encompasses contextual factors such as the clinical environment and organization of care delivery.^33^

Evolving paradigms in dentistry have shifted emphasis from extensive restorative procedures toward prevention and aesthetics. Older dentists (>70 years) demonstrated less nuanced understanding of multifactorial staining and lower patient engagement, consistent with educational models emphasizing more invasive protocols.^34^

Although generalizations about curricular priorities should be made cautiously, public universities in Brazil tend to emphasize evidence-based practice, research-informed education, and outreach activities, which can foster a deeper understanding of complex clinical issues,^35^ including the multifactorial etiology, assessment, and conservative management of dental discoloration. In contrast, private programs, particularly those with a market-driven focus, may place greater emphasis on procedural application, potentially leaving some theoretical and nuanced aspects less explored. Consistent with these trends, dentists trained in private institutions generally performed worse across most outcomes than graduates from public universities.

These factors may explain why dentists with graduate training, whether lato sensu or stricto sensu, in Operative Dentistry, Pediatric Dentistry, or Orofacial Harmonization show a stronger understanding of dental discoloration and non- or microinvasive management. Each specialty provides a unique yet interrelated perspective that enhances aesthetic care, and the emphasis on continuing education and technology in graduate programs, approached differently in lato versus stricto sensu formats, further supports clinical proficiency and success.^36^

Notably, dentists with >20 years of clinical experience but <70 years old tend to exercise greater caution, favoring techniques that preserve tooth structure whenever possible. With experience, emphasis shifts toward safeguarding natural teeth and considering the long-term impact of treatment choices on tooth longevity.^37^ At the same time, these practitioners recognize that more invasive measures may occasionally be required to achieve durable and satisfactory outcomes, particularly for complex or persistent discolorations or when non- or microinvasive approaches prove insufficient. Experience enhances the critical appraisal of treatment options, deepening their understanding of the benefits and limitations inherent to each approach.^38^

Beyond clinical experience, stricto sensu graduate training refines diagnostic acuity for dental discoloration by integrating theoretical depth, advanced technical skill, technological familiarity, and research literacy.^39^ These capabilities support more accurate depth estimation via transillumination and a better interpretation of porosity-related discolorations.

Finally, consultation of official statistics from the Federal Council of Dentistry^40^ confirms that the respondent profile closely reflects the national distribution of dentists, supporting the representativeness of our findings. Brazilian dentists are predominantly young and female (>60%), reflecting early career entry, the large number of dental schools in the country, and the progressive feminization of the profession. Most are concentrated in large urban centers of the Southeast, which also host the nation’s highest density of dental schools. The strong participation from São Paulo likely reflects the fact that, to the best of our knowledge, only its regional council disseminates surveys via direct email to registrants. Although private institutions numerically dominate dental training in Brazil, most respondents were graduates from public universities more than 20 years ago, indicating a cohort more deeply engaged with research, scientific inquiry, and academic communication via email. Specialization remains a key axis of professional development, improving care quality and service scope. Notably, the most frequently pursued career paths are not aesthetics-oriented, but Orthodontics, Implantology, Endodontics, Prosthodontics, and Periodontics. While many dentists work in clinics and service networks, private practice remains the predominant mode of professional activity.^40^

Across all professional backgrounds, dentists must stay informed about safe, and preferably non- or microinvasive, approaches to dental discoloration. Optimal outcomes rely on robust knowledge and sharpened clinical skills. This study provides evidence on dentists’ comprehension of discoloration and how training and professional trajectory affect clinical decision-making processes. Concurrently, certain methodological considerations must be acknowledged. As a cross-sectional online survey, it is susceptible to recall and social-desirability bias. The instrument, though tailored for this investigation, has not been previously validated, which may affect cross-study comparability. Dissemination heterogeneity across Regional Dental Councils may have influenced sample reach. Cross-sectional designs support the interpretation of associations rather than causation.

Despite these considerations, the results reveal essential gaps in the diagnosis, assessment, and management of discoloration, highlighting the need for continued professional development and for evidence-based non- or microinvasive approaches.

## Conclusion

Brazilian dentists demonstrated a good understanding of the etiology of dental discoloration and the methods used to assess it. However, their knowledge of treatment options and the extent of tissue removal remains moderate. Nonetheless, non- and microinvasive approaches for managing dental discoloration are widely recognized and frequently recommended as viable alternatives. Overall, the global outcome reflects only a moderate level of knowledge on the topic.

Dentists who graduated from public institutions and those with graduate training in Operative Dentistry, Pediatric Dentistry, or Orofacial Harmonization have shown the most thorough mastery of the subject. Targeted efforts are needed to improve awareness and dissemination of current evidence on non- and microinvasive strategies for treating dental discoloration, including their indications, advantages, limitations, and tissue preservation, particularly among professionals without training from public universities or graduate specialization in these fields.
